# Risk factors for adverse drug reactions in pediatric inpatients: A cohort study

**DOI:** 10.1371/journal.pone.0182327

**Published:** 2017-08-01

**Authors:** Paulo Henrique Santos Andrade, Iza Maria Fraga Lobo, Wellington Barros da Silva

**Affiliations:** 1 Universidade Federal de Sergipe, São Cristóvão, Sergipe, Brazil; 2 Hospital Universitário de Sergipe, Aracaju, Sergipe, Brazil; University of Catanzaro, ITALY

## Abstract

**Purpose:**

The present study aims to identify the risk factors for adverse drug reactions (ADR) in pediatric inpatients.

**Methods:**

A prospective cohort study in one general pediatric ward in a hospital in Northeast Brazil was conducted in two stages: the first stage was conducted between August 17^th^ and November 6^th^, 2015, and the second one between March 1^st^ and August 25^th^, 2016. We included children aged 0–14 years 11 months hospitalized with a minimum stay of 48 hours. Observed outcomes were the ADR occurrence and the time until the first ADR observed. In the univariate analysis, the time to the first ADR was compared among groups using a log-rank test. For the multivariate analysis, the Cox regression model was used.

**Results:**

A total of 173 children (208 admissions) and 66 ADR classified as “definite” and “probable” were identified. The incidence rate was 3/100 patient days. The gastro-intestinal system disorders were the main ADR observed (28.8%). In addition, 22.7% of the ADR were related to antibacterials for systemic use and 15.2% to general anesthesia. Prior history of ADR of the child [hazard ratio (HR) 2.44; 95% confidence interval (CI) 1.19–5.00], the use of meglumine antimonate (HR 4.98; 95% CI 1.21–20.54), antibacterial for systemic use (HR 2.75; 95% CI 1.08–6.98) and antiepileptic drugs (HR 3.84; 95% CI 1.40–10.56) were identified risk factors for ADR.

**Conclusions:**

We identified as risk factors the prior history of ADR of the child and the use of meglumine antimonate, antibacterial for systemic use and antiepileptic drugs.

## 1 Introduction

The vulnerability of children to Adverse Drug Reactions (ADR) into the hospital environment, the risk factors associated with the occurrence of these events and the association of these events with death are issues that awaken the interest of the scientific and healthcare communities around the world [1–8]. Furthermore, patient safety, high cost of these events, off-label use and absence of clinical research about the drug use in this age group enhances the interest of studying such population [1–9]. However, there is no consensus on which factors are associated with ADR in pediatric inpatient. Until now, the only significant association observed among the studies is polypharmacy [8, 10].

In this perspective, some studies have methodological biases, which may affect the results found. The lack of documentation and underreporting ADR, for example, may compromise the studies that restrict their data collection to the medical records and pharmacovigilance databases respectively [11].

Further prospective studies, with patient follow-up and patient ADR observation on the hospital bed, seem to be necessary for the investigation of ADR and their risk factors. Therefore, it is important to interview the patient and/or their family during data collection to obtain more information. In addition, it is necessary to assess the risk factors for ADR in a less holistic, and more specific and predictable way. Thus, the present study aims to identify the risk factors for ADR in pediatric inpatients.

## 2 Methods

The study was written based on the Strengthening the Reporting of Observational Studies in Epidemiology (STROBE; S1 Table) statement [12].

### 2.1 Study design

This was a prospective cohort study conducted in one general pediatric ward of a public teaching hospital of Sergipe, in Northeast Brazil. This hospital has 123 beds, and the chosen ward (which has 19 beds) involves several different pediatric specialties (Pneumonology, Gastroenterology, Cardiology, Neurology, Nephrology and Surgery).

A pilot study was conducted and incorporated into the data summary. Therefore, the periods of recruitment, follow-up, and data collection were conducted in two stages, the first stage, which was the pilot study, between August 17^th^ and November 6^th^, 2015, and the second one between March 1^st^ and August 25^th^, 2016. In addition, the exposure period occurred between the first administration of any medication and hospital discharge.

### 2.2 Eligibility criteria

We included children aged 0–14 years 11 months admitted in the pediatric ward with a minimum stay of 48 hours. The age range was defined based on the maximum age allowed for admission to the ward. The minimum stay was 48 hours due to the occurrence of significant sampling losses in a minor hospital stay, since it was not possible to include patients who were admitted on weekends or stayed for a period less than 24 hours. The children patients that were not discharged from the hospital within the study period, which was until November 6^th^, 2015 in the first stage, and until August 25^th^, 2016 in the second stage, were excluded once they were not followed during all hospital stay. In addition, we excluded the children that were not in use of drugs during hospitalization. Although the children admitted due to ADR have been included in this study, these ADR were not included in the statistical analysis.

### 2.3 Methods of selection, follow-up and data collection

One pharmacist specialized in epidemiology who had four years of experience in pharmacovigilance and ADR research conducted the selection, recruitment, follow-up, and data collection, daily and prospectively, except on weekends.

Children were recruited until 48 hours after admission to the pediatric ward. Follow-up was initiated after written informed consent of the guardian, and verbal consent of children who were aged over seven years old.

The follow-up and data collection included: record review (drug prescriptions, multidisciplinary records, laboratory tests), interview with the child and / or their family, discussion with the health care team (doctors, nurses, pharmacists and psychologists), observation of apparent clinical events on the bed (e.g., cutaneous and vascular events), and assessment of the ADR spontaneous reports.

The data collection was conducted from formularies adapted of the pharmacovigilance service from the present hospital [13]. The information on suspected ADR were collected at all follow-up stages. From the medical records were collected the information about the child (name, sex, age, date of birth and weight), admission diagnosis, drugs administered during hospitalization and its dosage, and clinical-laboratorial changes. During the interview was collected the prior history of ADR of the child and family member of first and second degree occurred before admission and the drugs for continued use at home. The prior history of ADR was defined as any ADR, independent of the degree of gravity, described by the child or family member during the first interview, which didn't have any association with the underlying disease present at the time the event occurred and which occurred prior to hospitalization, or in a previous hospitalization.

We excluded from the analysis the following drugs: intravenous hydration fluids, topical drugs, rectal washouts, blood products, oxygen therapy, parenteral nutrition and dextrose injection concentrate. These drugs were excluded in order to reduce bias, since a failure was observed in the data collection of such drugs. However, we included the following electrolytes: potassium chloride injection concentrate, sodium chloride injection, hypertonic concentration greater than 0.9%, magnesium sulfate injection, calcium gluconate 10%, because in Brazil these drugs are considered potentially dangerous drugs.

### 2.4 ADR diagnostic criteria

The main outcome assessed in this cohort study was the ADR occurrence. ADR was defined as "a response to a drug which is noxious and unintended, and which occurs at doses normally used in man for the prophylaxis, diagnosis, or therapy of disease, or for the modification of physiological function" [14]. Thus, we included any event associated with a drug, which was used at doses normally used in man, not exceeding the age-specific dose, even though this event is related to a medication error, "off-label" use and drug-drug interactions.

The term "off-label" was defined as the use of drugs that were contraindicated for a given age range. We emphasize that all drugs related to the “off-label” use were administered at doses normally used in man. In both concepts, dosage information was obtained from Drugs.com [15].

To assess the causality and severity of the suspected adverse drug events (ADE), the Naranjo [16] and Hartwig [17] algorithms were used, respectively. Causality and severity assessment was undertaken by a single assessor, a pharmacist specialized in epidemiology.

We included in this study only the ADR classified as “definite” (≥ 9 points on the scale of Naranjo) and “probable” (5–8 points on the scale of Naranjo). The reactions classified as possible (1–4 Naranjo scale points) were excluded due to the lesser degree of association with the event. In addition, the reactions classified as possible are questionable associations due to the fact that they can be classified after the compliance of only one item of the scale of Naranjo. The exclusion of these reactions also reduced the bias related to causality assessment, which occurred due to the absence of another professional from the area during the causality classification.

After this assessment, the events were classified according to the Adverse Reaction Terminology (ART) [18], the suspected drugs based on the Anatomical Therapeutic Chemical (ATC) [19] and the diagnosis based on the International Classification of Diseases version 10 (ICD 10) [20], all of the World Health Organization (WHO).

### 2.5 ADR incidence

The ADR overall incidence was calculated based on the number of admissions and number of children. For this calculation, we divided: the total number of admissions (or child’s number) with at least one ADR, by the total number of admissions (or child’s number), who used at least one drug. In addition, the ADR incidence rate was determined from the division of the number of ADR new cases and the sum of the days of hospital stay of children included (patient-day), who used at least one drug. The confidence intervals at 95% (95% CI) for all incidence calculations were calculated.

In the present study, the ADR recurrence was included as a new case; the ADR that occurred at the same time, which were assigned to the same drug, were calculated as a single reaction (e.g. nausea and vomiting occurring at the same time, due to the administration of only one drug, were considered as a single reaction); and the patient-day was calculated from the subtraction between discharge and admission date at the hospital plus one day.

### 2.6 Risk factor analysis

We evaluated as ADR risk factors the variables:

Dichotomous (yes, no): Cystic fibrosis (ICD 10 E84), prior history of ADR of the child and their family of first and second degree, received a general anesthesia, received a meglumine antimonate, received a metamizole sodium, received an antibacterial for systemic use, received an antihistamine drug, received an antiepileptic drug, use of omeprazole and clonazepam on the same day and gender (male, female); and

Continuous: age, number of drugs administered (number of drugs administered during the hospital stay), number of new drugs administered after admission (drugs administered during the hospital stay excluding drugs of continued use administered at home one day before admission), number of intravenous drugs administered.

These risk factors were selected based on a systematic review [21] conducted prior to the present study. The other included factors were variables that stood out in the studied population or represented the most used drug classes for the treatment of diseases described in the review as risk factors.

In addition, some of these variables have also been evaluated in specific reactions such as vomiting and nausea, skin and appendages disorders, somnolence and diarrhea. To reduce information bias in the present study, we included the administered drugs instead of prescription drugs.

### 2.7 Statistical analysis

The data were organized using the Microsoft Excel^®^ (Microsoft Corporation) and the statistical analysis was carried out using the statistical software IBM SPSS statistics 20.0. In the univariate analysis, the time to the first ADR was compared among groups using a log-rank test. For the multivariate analysis, the Cox regression model was used, and the Enter method was applied. Due to the clinical importance, all variables analyzed were included in the multivariate model. Results are giving in terms of the hazard ratio (HR) and 95% CI. For all tests, *p* < 0.05 was selected as the level for statistical significance. Statistically significant risk factors in the multivariate analysis were plotted on a cumulative hazard graph. As there were no missing data, no imputation techniques were required.

### 2.8 Ethical approval

The following study was approved by the ethics committee (CAE: 49160315.5.0000.5546). All procedures performed in studies involving human participants were in accordance with the ethical standards of the institutional and/or national research committee and with the 1964 Helsinki declaration and its later amendments or comparable ethical standards. Written informed consent of the guardian, and verbal consent of children who were aged over seven years old were obtained from all individual participants included in the study.

## 3 Results

**Fig 1:**
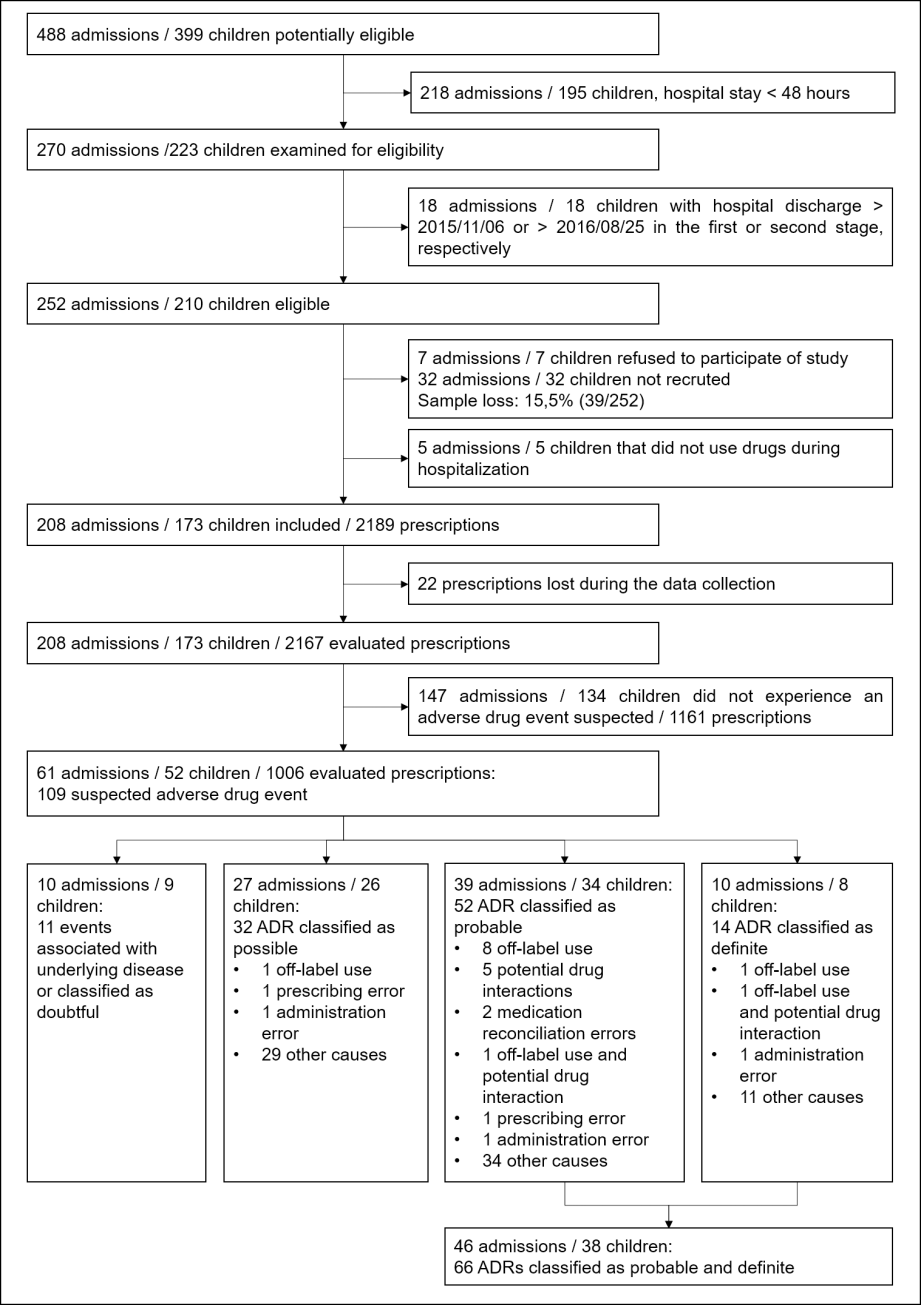
Flowchart outlining the number of admissions included and children in the study. ADR: adverse drug reaction. NOTE: the term "off-label" was defined as the use of drugs that are contraindicated for a given age range, based on information available in the Drugs.com [15].

A total of 208 admissions of 173 children was included in this study and followed during all hospital stay (Fig 1). In addition, 2167 prescriptions were assessed during data collection. Of the 2163 drugs (active ingredient) prescribed, 12.5% (271) were not administered. Concerning children by admission, five (2.9%) children had three admissions, 25 (14.5%) had two admissions and other 143 (82.6%) had one admission. During the follow-up, only two children went to the Intensive Care Unit (ICU) of present hospital which one (0.6%) of them was due to ADR (Table 1). In this case, one error occurred due to the inappropriate administration of the vancomycin.

**Table 1:** Assessment of severity using the Hartwig severity scale.

Severity level	Description	Frequency of the ADR at each severity level	
		N	n/66 (%)
1	An ADR occurred but no change in treatment with suspected drug	19	28.8
2	The ADR that required treatment with the suspected drug; will be withheld, discontinued, or otherwise changed. No antidote or other treatment required. No increase in length of stay.	14	21.2
3	The ADR that required treatment with the suspected drug will be withheld, discontinued, or otherwise changed, and/or an antidote or other treatment. No increase in length of stay.	25	37.9
4	Any Level 3 ADR that increases the length of stay by at least one day or the ADR was the reason for admission.	7	10.6
5	Any Level 4 ADR that requires intensive medical care.	1	1.5

ADR: adverse drug reaction.

The median of hospital stay in the pediatric ward was eight days [interquartile range 1 and 3 (IQR) 4–14 days, amplitude 2–88 days]. The patient-day was 2171 days. The mean age was 4.8 years (standard deviation (σ) 4.19 years, amplitude 0 to 14 years), 95 (54.9%) were male. The median of the number of drugs administered was seven drugs (IQR 4–12 drugs, amplitude 1–33 drugs). The median of the new drugs administered after admission was six drugs (IQR 3–11 drugs, amplitude 0–30 drugs). In addition, the median of the number of intravenous drugs administered was three (IQR 1–6 drugs, amplitude 0–22 drugs).

### 3.1 Frequency and characteristics of ADRs

In total, 109 suspected ADE of 52 children were assessed. Of this total, 14 (12.8%) ADR was classified as “definite”, 52 (47.7%) as “probable”, 32 (29.4%) as “possible” and 11 (10.1%) were associated with an underlying disease or classified as “doubtful”. We included in the study all “definite” and “probable” ADR, in the total number of 66 ADR of 38 children (46 admissions) (Fig 1). Of these 46 admissions, one child (2.2%) had four ADR, 11 (23.9%) children had three ADR, 10 (21.7%) children had two ADR and 24 (52.2%) children had only one ADR.

The gastro-intestinal system disorders (WHO-ART 0600) was the main ADR observed and corresponded to 19 (28.8%) of the included ADR. Somnolence was the most frequently observed ADR, 11 (16.6%). In addition, 15 (22.7%) ADR were related to antibacterials for systemic use (WHO-ATC J01). More information about the ADR types, drugs implicated in ADR and severity level of the ADR can be observed in S2 Table.

### 3.2 Incidence of ADRs during hospitalization

The overall incidence of “definite” and “probable” ADR in the present cohort study based on admissions was 22.1% [(46/208); 95% CI 16.7 to 28.0] and 21.9% [(38/173); 95% CI 16.1 to 28.5] when based on numbers of children. The incidence rate was 3/100 patient day [66/2171; 95% CI 2.3 to 3.8/100 patient day].

### 3.3 Risk factor analysis

**Fig 2:**
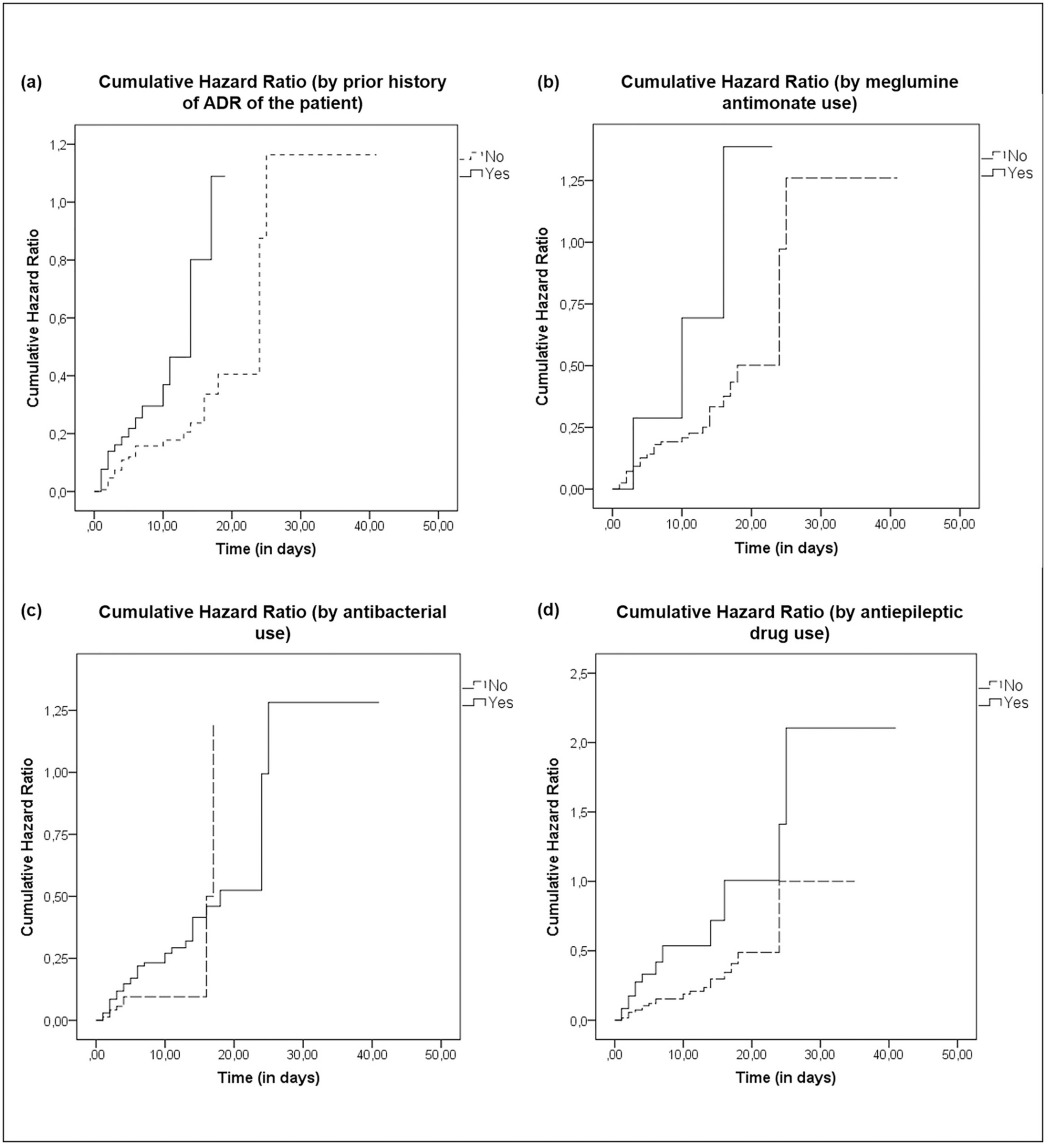
Cumulative hazard ratio curves by categorical time invariant risk factor by: (a) prior history of ADR of the patient, (b) meglumine antimonate use, (c) antibacterial use and (d) antiepileptic drug use.

Results of the univariate and multivariate analysis are shown in Table 2, Fig 2. The main reaction types were described below with other factors related to them.

**Table 2:** Univariate and multivariate analysis by categorical time invariant risk factor.

Variables		ADR occurred		Univariate	Multivariate	
				Log-rank statistic	Cox Regression	
		S	N	*p*-value	HR (95% CI)	*p*-value
Gender	Female	24	71	0.37	0.74 (0.38–1.43)	0.38
	Male	22	91			
Age on admission (in years)				0.95	0.98 (0.90–1.06)	0.64
Prior history of ADR of the patient	No	29	125	0.007*	1	0.01*
	Yes	17	37		2.44 (1.19–5.00)	
Prior history of ADR of the family of first and second degree	No	37	126	0.78	1	0.67
	Yes	9	36		1.18 (0.53–2.62)	
Received a GA	No	36	137	0.15	1	0.12
	Yes	10	25		2.59 (0.77–8.68)	
Received a meglumine antimonate	No	43	161	0.10	1	0.02*
	Yes	3	1		4.98 (1.21–20.54)	
Received a metamizole sodium	No	22	88	0.51	1	0.07
	Yes	24	74		0.48 (0.22–1.07)	
Received an antibacterial for systemic use	No	8	65	0.18	1	0.03*
	Yes	38	97		2.75 (1.08–6.98)	
Received an antihistamine drug	No	42	156	0.72	1	0.72
	Yes	4	6		1.26 (0.34–4.65)	
Received an antiepileptic drug	No	33	150	0.006*	1	0.009*
	Yes	13	12		3.84 (1.40–10.56)	
Use of omeprazole and clonazepam on the same day	No	41	162	0.01*	1	0.18
	Yes	5	0		2.39 (0.66–8.63)	
Cystic fibrosis (ICD 10 E84)	No	43	157	0.74	1.97 (0.48–8.09)	0.34
	Yes	3	5			
Number of drugs administered				0.09	0.88 (0.72–1.07)	0.22
Number of new drugs administered after admission				0.04*	0.96 (0.77–1.19)	0.73
Number of intravenous drugs administered				0.40	1.16 (0.99–1.36)	0.06

ADR: adverse drug reaction. CI: confidence interval. GA: general anesthesia. HR: hazard ratio. ICD: International Classification of Diseases.

*p-value < 0.05.

#### 3.3.1 Risk factor for vomiting and nausea

A total of 12 events of vomiting and nausea were observed. Seven of these events (58.3%) were related to surgery (five to General anesthesia, two to preoperative preparation). Two (16.6%) events were related to a probable inadequate administration of the drugs and one (8.3%) with a medication reconciliation error (S3 Table).

#### 3.3.2 Risk factor for skin and appendages disorders

We observed 11 skin and appendages disorders. Of this total, five events (45.4%) were related with antibacterials for systemic use (WHO-ATC J01). Among the events associated to antibacterials, one occurred due to an administration error (S4 Table).

#### 3.3.3 Risk factor for somnolence

In total, 11 events of somnolence were observed and all of them occurred early in drug therapy. Seven of these events (63.6%) were related with antiepileptics (WHO-ATC N03) or analgesics (WHO-ATC N02) drugs, from which, three were related to drug interactions and one with medication reconciliation error. Alterations in the dosage (dose and route) of antiepileptic drugs were factors that influenced the association of this drug class with somnolence (S5 Table).

#### 3.3.4 Risk factor for diarrhoea

Eight events of diarrhoea were observed. Four of these events (50.0%) were related with antibacterials for systemic use, after a minimum of six days of administration. In addition, two of these events were related to *Clostridium difficile* due to the improvement of this event has been associated with the administration of metronidazole.

## 4 Discussion

To our knowledge, this is the third Brazilian study to prospectively evaluate risk factors for ADR in pediatric inpatients [5, 6]. This is the first Brazilian study with these criteria, which used survival analysis for risk assessment. Also, it is the second study with these criteria worldwide, which used survival analysis for this risk assessment [1–7] (S6 Table).

### 4.1 Frequency and characteristics of ADRs

Regarding the observed ADR, it is important to highlight that the vast majority of them were ADR perceptible to the naked eye. The communication barrier between children and adults may have influenced these results since different language skills or stages of development do not allow children to report symptoms. Thus, some events may not be observed or even be interpreted as restlessness or lethargy.

As in the present study, other studies identified gastro-intestinal system disorders as the main ADR [2, 4]. In addition, among Brazilian studies, antibacterials for systemic use also prevailed among drugs related to ADR [5, 6], which in the present study were related to gastro-intestinal system disorders, skin and appendage disorders and application site disorders.

### 4.2 Incidence of ADRs during hospitalization

We observed that the overall incidence of ADR per patient identified in the present study (22.0%) is higher than the overall incidence of ADR observed in other studies with similar methodology, including one of the Brazilian studies (range 2.7%-17.7%) [1–4, 6, 7]. The incidence of the present study was only lower than the incidence of the Brazilian study conducted exclusively in an intensive care unit (ICU; 35.1%) [5]. In addition, this incidence is greater than the incidences (0,5–16,8%) found in two systematic reviews that included case-control studies [8,10].

As a justification, we observed that data collection and ADR identification in some studies did not include child and / or their family interview [1–5, 7]. For the same purpose, the studies used methods such as assessment of medical records [1–4], active search method based on triggers [5], or spontaneous reporting [7]. Therefore, the researchers of these studies may have disregarded underreporting of ADR [11, 22], or they may have disregarded some ADR which may have occurred, but that has not been identified due to the absence of specific triggers that could identify this ADR [5].

Regarding the information collected during the interview, we knew it might not be as trustworthy as the information reported in medical records, but we also knew the importance of this report for pharmacovigilance [23, 24]. Therefore, we conducted an investigation for each reported event. In addition, to reestablish this reliability, the investigation included discussions with professionals, assessment of medical records and causality assessment.

Other reasons, which influenced the ADR incidence, observed in these studies were [1–7] the definition or interpretation of the concept of ADR, causality assessment, inclusion or not of ADR classified as "possible" and environmental influences, since several hospitals around the world have been included, and some hospitals may have more effective safety tools for ADR prevention.

Concerning the study conducted exclusively in the ICU [5], we can state that the high incidence is something expected, due to the highest number of drugs administered in this population [25] and this variable have been considered a risk factor in all the referred studies [1–7].

In relation to defining and interpreting the concept of ADR, we observed that none of these studies associated any ADR to drug-drug interactions, administration errors, prescription errors and medication reconciliation [1–6], and that only one of them described a relation between ADR and off-label use [7]. However, another similar study associated these factors to ADE instead of the ADR.

This may be a fault in the present study; however, all events included were events arising from the usage of drugs at doses normally used in man, not exceeding the age-specific dose. Including the events related to medication errors, off-label use and drug-drug interactions. Thus, the concept of ADR in this study also fits the new European concept which defines ADR only as “a response to a medicinal product which is noxious and unintended” irrespective of the way of using medicines [26]. The new definition enlarges the WHO definition, including also ADR resulting from unauthorized uses of medicines, such as off-label use, overdose, abuse, misuse, and from medication errors [26].

The ADR included in the present study involved the use of: i) drugs administered, which were not prescribed; ii) prescription of a higher dose than the used at home; iii) use of untested drugs in the age group; iv) prescription of higher dose than the dose which was pre-established in discussion by medical specialists; or v) administration in shorter time than the time prescribed for the drug.

Another issue to be discussed is the use of the Naranjo algorithm for the causality assessment. In the present study, we showed the drugs with the highest causality scores. However, as in other studies [1–5], these drugs may not indeed be the drugs responsible for ADR, since there might be other factors that were not addressed by Naranjo and this would influence the classification.

When we used the Naranjo algorithm and only analysed the "probable" and "definite" ADR, we excluded events that could be associated with other causes such as the underlying disease, or the use of long-term peripheral venous access, in the case of phlebitis. However, if certain procedures were linked exclusively to the use of drugs, was this conduct the most appropriate, or would it be more appropriate to evaluate all ADE, as Eshetie and collaborators [27] and the European Pharmacovigilance Legislation?

### 4.3 Risk factors observed

#### 4.3.1 Prior history of ADR

Concerning the risk factors observed, we highlight the prior history of ADR of the child. Despite being an expected variable as a risk factor [28], it has not been evaluated in previous studies of similar methodology yet [1–7]. Our findings indicate that patients with a prior history of ADR were 2.44 times (HR 2.44; 95%CI 1.19–5.00; p = 0.01) more likely to develop an ADR than those who had no prior history.

Prior history of ADR is a relevant risk factor, since the record of allergy and other ADR in medical records is not always done, or the patient is not always asked about this prior history, a fact not only observed in the present hospital [29, 30]. Poor documentation on patients with a history of ADR may lead to re-exposure of the allergen or of the drugs with similar chemical structure causing the patient to experience the same ADR again.

Although some children had a new ADR which were different from that previously reported, or the same ADR associated with a new class of medication, there were children who had the same ADR of the same drug class previously reported. Therefore, poor documentation may have been a confounding factor that was not analyzed in the present study.

#### 4.3.2 Use of antibacterial for systemic use

Our findings indicate that patients who use antibacterials for systemic use were 2.75 times (HR 2.75; 95% CI 1.08–6.98) more likely to have an ADR than patients who did not use them. In similar studies, it is possible to observe that this drug class was the therapeutic groups most commonly associated with suspected ADR [1–3, 5–7]. However, one of these studies did not find a statistically significant association between antibiotic use and suspected ADR [6] and none of the other studies performed the analysis between these variables [1–3, 5, 7]. It is possible to observe in these studies that some of the factors identified were directly related to this drug class. Therefore, the use of antibacterial for systemic use may be a confounding factor not analyzed in these studies.

In one of these studies [1], for example, “certain infectious and parasitic disease” (ICD 10 A00-B99) were associated with suspected ADR, as well as in another study where the number of high-risk drugs, which included antibiotics, were associated with suspected ADR [3]. Therefore, we asked ourselves whether the analysis performed in these studies was the most adequate since these researchers analyzed the secondary risk factor instead of the primary risk factor that would be the use of antibacterials for systemic use.

#### 4.3.3 Use of antiepileptic drug

The use of antiepileptic drugs (HR 3.84; 95% CI 1.40–10.56) was a risk factor for ADR in pediatric inpatient. Similar to the antibacterial for systemic use, the antiepileptic drug was one of the main therapeutic groups most commonly associated with suspected ADR in similar studies [1–3, 5, 6]. Although the use of antiepileptic drugs has not been analyzed as a risk factor for ADR in these studies [1–7], the use of such drugs may be a confounding factor in the study of Rashed [3]. Therefore, Rashed and collaborators may have analyzed the secondary risk factor when considering the "diseases of the nervous system" (ICD 10 G00-G99), and the increase in the number of high-risk drugs prescribed, which included antiepileptic drugs, as risk factors [3].

#### 4.3.4 Use of meglumine antimonate

Among the risk factors observed in this cohort study, the use of meglumine antimonate was the highest risk of ADR (HR 4.98; 95% CI 1.21–20.54). As well as observed in the last two topics above, the use of meglumine antimonate was not assessed as a potential risk factor in similar studies [1–7]. Although few children used this medication, 75% (3) of them had ADR. The use of meglumine antimonate may not be studied by the majority of these studies due to its use for the treatment of leishmaniasis which is a common disease in developing countries.

### 4.4 Limitation

In the present study, we did not observe polypharmacy as a risk factor, which was identified in all similar publications previously cited [1–7], as well as other risk factors, which were not identified. All results presented here may have been influenced by the sample size assessed, and should not be interpreted as an absolute result. This is the main limitation of this work. In addition, causality assessment was undertaken by a single assessor. Assessment of the ADR cases by another individual, preferably from another discipline (e.g. doctor or nurse) would have been ideal. However, the present work brings novelties as new variables were analyzed, the conducted method and the analysis presented by specific groups of ADR aside from combined analysis of all the ADR. In addition, we identified specific risk factors for the main classes of ADR observed due to the evaluation of risk factors to be more appropriate for specific groups.

## 5 Conclusion

ADR are frequent events in pediatric inpatient. The gastro-intestinal system disorders and antibacterials for systemic use were the ADR and drug class most frequent, respectively. In the present study, we identified as risk factors for ADR the prior history of ADR of the patient, the use of meglumine antimonate, antibacterial for systemic use and antiepileptic drugs. Furthermore, we emphasized the importance of registering the prior history of ADR of the patient and maintaining greater vigilance during the administration period of these drugs. The health professionals should be vigilant and monitor, more closely, the patients who use these drugs and should pay attention to drug-drug interactions and changes in antiepileptic drugs dosage (e.g. dose and route) since that these were other factors that influenced the ADR occurrence.

## Supporting information

S1 Table STROBE Checklist for cohort studies. NA: not applicable. Suppl.: Supplement. *Give information separately for exposed and unexposed groups. Note: An Explanation and Elaboration article discusses each checklist item and gives methodological background and published examples of transparent reporting. The STROBE checklist is best used in conjunction with this article (freely available on the Web sites of PLoS Medicine at http://www.plosmedicine.org/, Annals of Internal Medicine at http://www.annals.org/, and Epidemiology at http://www.epidem.com/). Information on the STROBE Initiative is available at http://www.strobe-statement.org.
(PDF)

S2 Table Adverse drug reactions types, drugs and severity level observed. WHO-ART: Adverse Reaction Terminology, World Health Organization. WHO-ATC: Anatomical Therapeutic Chemical, World Health Organization. “or”: Same level of causality. “x”: drug-drug interaction. *The event occurred in the surgical center. †One of the events was classified as “definite” according to the Naranjo algorithm. ‡All events were classified as “definite” according to the Naranjo algorithm.
(PDF)

S3 Table Risk factors by univariate and multivariate analysis for vomiting and nausea. NOTE: Although there have been 12 events of nausea and vomiting. In this analysis only 11 were included, since one child had the same ADR twice. Therefore, only the first ADR was included in the analysis. ADR: adverse drug reaction. CI: confidence interval. GA: general anesthesia. HR: hazard ratio. *p-value < 0.05.
(PDF)

S4 Table Risk factors by univariate and multivariate analysis for skin and appendages disorders. ADR: adverse drug reaction. CI: confidence interval. GA: general anesthesia. HR: hazard ratio. ICD: International Classification of Diseases. *p-value < 0.05.
(PDF)

S5 Table Risk factors by univariate and multivariate analysis for somnolence. ADR: adverse drug reaction. CI: confidence interval. GA: general anesthesia. HR: hazard ratio. *p-value < 0.05.
(PDF)

S6 Table Comparison between the characteristics of the present cohort study and the characteristics of prospective cohort studies on risk factors for adverse drug reactions in pediatric inpatient. D50-D89: Diseases of the blood and blood-forming organs and certain disorders involving the immune mechanism; G00-G99: Diseases of the nervous system; ICU: Intensive care units. OI: overall incidence. NICU: neonatal intensive care units. PICU: pediatric intensive care units. WHO: World Health Organization; P00–P96: Certain conditions originating in the perinatal period. *the overall incidence was calculated by the author of this paper, from the sample size and patient with ADR number presented in the study. †the patient with ADR number was calculated by the author of this paper, from the overall incidence and sample size presented in the study. ‡Multivariate analysis.
(PDF)
