# Supplementary material for: Risk factors for adverse drug reactions in pediatric inpatients: A cohort study
Source: PLoS One. 2017 Aug 1;12(8):e0182327. doi: 10.1371/journal.pone.0182327 (PMC5538648; doi:10.1371/journal.pone.0182327)
Supplement: S2 Table — WHO-ART: Adverse Reaction Terminology, World Health Organization. WHO-ATC: Anatomical Therapeutic Chemical, World Health Organization. “or”: Same level of causality. “x”: drug-drug interaction. *The event occurred in the surgical center. †One of the events was classified as “definite” according to the Naranjo algorithm. ‡All events were classified as “definite” according to the Naranjo algorithm. (PDF) [file pone.0182327.s002.pdf]

S2 Table. Adverse drug reactions types, drugs and severity level observed.

System-organ classes	WHO-ART	Main Event	Drugs	WHO-ATC	Severity level
Gastro-intestinal system disorders (19)	0600	Vomiting (10)	Macrogol, combinations (2)†	A06AD65	1, 3
			Metamizole sodium (1)	N02BB02	3
			Sodium chloride (1)	B05XA03	2
			Meglumine antimonate (1)	P01CB01	1
			Sulfamethoxazole and trimethoprim (1)	J01EE01	1
			GA (4)	-	1, 1, 3, 3
		Diarrhoea (8)	Amikacin x Cefepime x Ciprofloxacin (1)	J01GB06 x J01DE01 x J01MA02	4
			Amoxicillin and enzyme inhibitor (1)	J01CR02	3
			Azithromycin (1)	J01FA10	3
			Ceftriaxone x Clindamycin (1)	J01DD04 x J01FF01	3
			Domperidone (1)	A03FA03	3
			Ferrous sulfate (1)	B03AA07	2
			Lactulose (1)	A06AD11	2
			Macrogol, combinations (1)	A06AD65	2
		Nausea (1)	GA (1)	-	1
Psychiatric disorders (12)	0500	Somnolence (11)	Phenobarbital (3)	N03AA02	2
			Dexchlorpheniramine (2)	R06AB02	1
			Clonazepam x Phenobarbital (2)	N03AE01 x N03AA02	2
			Morphine (1)	N02AA01	2
			Omeprazole x Clonazepam (1)	A02BC01 x N03AE01	1
			Ketoprofen (1)	M01AE03	1
			GA (1)	-	1
		Agitation (1)	Fenoterol (1)‡	R03CC04	3
Skin and appendages disorders (9)	0100	Rash erythematous (3)	Azithromycin (1)	J01FA10	3
			Furosemide (1)	C03CA01	3
			GA (1)	-	3

S2 Table. Continued

System-organ classes	WHO-ART	Main Event	Drugs	WHO-ATC	Severity level
Skin and appendages disorders (9)	0100	Pruritus (2)	Methylprednisolone (1)	H02AB04	3
			GA (1)	-	1
		Rash erythematous and Pruritus (2)	Cefepime (1)‡	J01DE01	3
			Ceftriaxone (1)	J01DD04	3
		Angioedema (1)	Metamizole sodium (1)	N02BB02	3
		Rash erythematous and Angioedema (1)	Metamizole sodium (1)‡	N02BB02	3
Metabolic and nutritional disorders (6)	0800	Hypoglycaemia (4)	Insulin (3)‡	A10AE01	4
			Glibenclamide x Insulin (1)‡	A10BB01 x A10AE01	3
		Hypokalaemia (2)	Amphotericin B (1)	J02AA01	3
			Furosemide (1)	C03CA01	3
Application site disorders (5)	1820	Injection site inflammation (2)	Ceftriaxone (2)†	J01DD04	2
		Injection site pain (2)	Ceftriaxone (2)	J01DD04	1
		Application site oedema and pain (1)	Sodium chloride or Potassium chloride (1)	B05XA03 or B05XA01	3
Vascular (extracardiac) disorders (3)	1040	Flushing (3)	Methylprednisolone (2)	H02AB04	1
			Oxybutynin (1)‡	G04BD04	1
Respiratory system disorders (3)	1100	Coughing (1)	Ipratropium bromide (1)‡	R03BB01	4
		Respiratory depression (2)*	GA (2)	-	3
Body as a whole - general disorders (2)	1810	Oedema (1)	Hydrocortisone (1)	H02AB09	2
		Abdominal pain (1)	Albendazole (1)	P02CA03	1
Central and peripheral nervous system disorders (1)	0410	Convultions (1)	Hydrocortisone (1)	H02AB09	3
Liver and biliary system disorders (1)	0700	Hepatic enzymes increased (1)	Meglumine antimonate (1)	P01CB01	1
Resistance mechanism disorders (1)	1830	Hypotension (1)	Tobramycin (1)‡	J01GB01	2
White cell and RES disorders (1)	1220	Granulocytopenia (1)	Meglumine antimonate (1)	P01CB01	4

S2 Table. Continued

System-organ classes	WHO-ART	Main Events	Drugs	WHO-ATC	Severity level
Skin and Appendages Disorders and Respiratory system disorders (1)	0100 and 1100	Rash erythematous and coughing (1)	Meropenem (1)‡	J01DH02	4
Body as a whole - general disorders, Musculo-skeletal system disorders, and Gastro-intestinal system disorders (1)	1810, 0200 and 0600	Fever, Arthralgia, vomiting e abdominal pain (1)	Meglumine antimonate (1)	P01CB01	3
Skin and appendages disorders and central, Resistance mechanism disorders, Heart rate and rhythm disorders and Central & peripheral nervous system disorders (1)	0100, 1830, 1030 and 0410	Red Man Syndrome, Cyanosis, Bradycardia and Convulsions (1)	Vancomycin (1)‡	J01XA01	5

WHO-ART: Adverse Reaction Terminology, World Health Organization. WHO-ATC: Anatomical Therapeutic Chemical, World Health Organization.

“or”: Same level of causality. “x”: drug-drug interaction.

*The event occurred in the surgical center.

†One of the events was classified as “definite” according to the Naranjo algorithm.

‡All events were classified as “definite” according to the Naranjo algorithm.
