# Supplementary material for: Risk factors for adverse drug reactions in pediatric inpatients: A cohort study
Source: PLoS One. 2017 Aug 1;12(8):e0182327. doi: 10.1371/journal.pone.0182327 (PMC5538648; doi:10.1371/journal.pone.0182327)
Supplement: S3 Table — NOTE: Although there have been 12 events of nausea and vomiting. In this analysis only 11 were included, since one child had the same ADR twice. Therefore, only the first ADR was included in the analysis. ADR: adverse drug reaction. CI: confidence interval. GA: general anesthesia. HR: hazard ratio. *p-value < 0.05. (PDF) [file pone.0182327.s003.pdf]

S3 Table. Risk factors by univariate and multivariate analysis for vomiting and nausea.

Variables				Univariate	Multivariate	
		ADR occurred		Log-rank statistic	Cox Regression	
		S	N	*p*-value	HR (95% CI)	*p*-value
Gender	Female	5	90	0.91	1	0.82
	Male	6	107		0.85 (0.21-3.47)	
Age on admission (in years)				0.03*	1.12 (0.93-1.34)	0.21
Prior history of ADR of the patient	No	7	146	0.49	1	0.30
	Yes	4	51		2.18 (0.49-9.57)	
Prior history of ADR of the family of first and second degree	No	8	155	0.58	1	0.09
	Yes	3	42		3.66 (0.78-17.08)	
Received a GA	No	5	167	<0.001*	1	0.004*
	Yes	6	30		16.00 (2.42-105.51)	
Received a meglumine antimonate	No	9	195	0.009*	1	0.007*
	Yes	2	2		20.89 (2.30-189.70)	
Number of drugs administered				0.18	1.08 (0.77-1.51)	0.64
Number of new drugs administered after admission				0.002*	0.91 (0.62-1.33)	0.64
Number of intravenous drugs administered				0.02*	1.03 (0.82-1.29)	0.78

NOTE: Although there have been 12 events of nausea and vomiting. In this analysis only 11 were included, since one child had the same ADR twice. Therefore, only the first ADR was included in the analysis.

ADR: adverse drug reaction. CI: confidence interval. GA: general anesthesia. HR: hazard ratio.

*p-value < 0.05.
