# Supplementary material for: Risk factors for adverse drug reactions in pediatric inpatients: A cohort study
Source: PLoS One. 2017 Aug 1;12(8):e0182327. doi: 10.1371/journal.pone.0182327 (PMC5538648; doi:10.1371/journal.pone.0182327)
Supplement: S4 Table — ADR: adverse drug reaction. CI: confidence interval. GA: general anesthesia. HR: hazard ratio. ICD: International Classification of Diseases. *p-value < 0.05. (PDF) [file pone.0182327.s004.pdf]

S4 Table. Risk factors by univariate and multivariate analysis for skin and appendages disorders.

Variables		ADR occurred		Univariate	Multivariate	
				Log-rank statistic	Cox Regression	
		S	N	*p*-value	HR (95% CI)	*p*-value
Gender	Female	4	91	0.53	1	0.64
	Male	6	107		1.39 (0.33-5.82)	
Age on admission (in years)				0.87	0.92 (0.76-1.12)	0.44
Prior history of ADR of the patient	No	5	147	0.06	1	0.06
	Yes	5	51		5.13 (0.91-28.78)	
Prior history of ADR of the family of first and second degree	No	8	155	0.95	1	0.60
	Yes	2	43		0.57 (0.07-4.53)	
Received a GA	No	7	164	0.25	1	0.03*
	Yes	3	34		11.49 (1.26-104.16)	
Received a metamizole sodium	No	4	101	0.98	1	0.68
	Yes	6	97		1.40 (0.27-7.29)	
Received an antibacterial for systemic use	No	3	70	0.43	1	0.39
	Yes	7	128		0.46 (0.07-2.75)	
Cystic fibrosis (ICD 10 E84)	No	8	192	0.02*	1	0.10
	Yes	2	6		7.56 (0.66-86.63)	
Number of drugs administered				0.40	0.92 (0.66-1.28)	0.63
Number of new drugs administered after admission				0.81	1.07 (0.70-1.63)	0.75
Number of intravenous drugs administered				0.78	0.89 (0.64-1.23)	0.49

ADR: adverse drug reaction. CI: confidence interval. GA: general anesthesia. HR: hazard ratio. ICD: International Classification of Diseases.

*p-value < 0.05.
