# Supplementary material for: Risk factors for adverse drug reactions in pediatric inpatients: A cohort study
Source: PLoS One. 2017 Aug 1;12(8):e0182327. doi: 10.1371/journal.pone.0182327 (PMC5538648; doi:10.1371/journal.pone.0182327)
Supplement: S5 Table — ADR: adverse drug reaction. CI: confidence interval. GA: general anesthesia. HR: hazard ratio. *p-value < 0.05. (PDF) [file pone.0182327.s005.pdf]

S5 Table. Risk factors by univariate and multivariate analysis for somnolence.

Variables		ADR occurred		Univariate	Multivariate	
				Log-rank statistic	Cox Regression	
		S	N	*p* -value	HR (95% CI)	*p* -value
Gender	Female	7	88	0.24	1	0.25
	Male	4	109		0.40 (0.08-1.88)	
Age on admission (in years)				0.96	1.10 (0.88-1.36)	0.38
Prior history of ADR of the patient	No	9	142	0.43	1	0.51
	Yes	2	55		0.56 (0.09-3.22)	
Prior history of ADR of the family of first and second degree	No	8	155	0.64	1	0.75
	Yes	3	42		1.28 (0.27-5.90)	
Received a GA	No	10	162	0.55	1	0.84
	Yes	1	35		0.77 (0.05-10.12)	
Received an antihistamine drug	No	9	186	0.06	1	0.01*
	Yes	2	11		11.85 (1.61-86.80)	
Received an antiepileptic drug	No	5	176	<0.001*	1	<0.001*
	Yes	6	21		35.36 (6.40-195.47)	
Use of omeprazole and clonazepam on the same day	No	10	193	0.34	1	0.70
	Yes	1	4		1.66 (0.12-22.98)	
Number of drugs administered				0.94	0.41 (0.21-0.79)	0.008*
Number of new drugs administered after admission				<0.001*	1.96 (1.04-3.71)	0.03*
Number of intravenous drugs administered				0.07	1.25 (1.01-1.54)	0.04*

ADR: adverse drug reaction. CI: confidence interval. GA: general anesthesia. HR: hazard ratio.

*p-value < 0.05.
