# Supplementary material for: Risk factors for adverse drug reactions in pediatric inpatients: A cohort study
Source: PLoS One. 2017 Aug 1;12(8):e0182327. doi: 10.1371/journal.pone.0182327 (PMC5538648; doi:10.1371/journal.pone.0182327)
Supplement: S6 Table — D50-D89: Diseases of the blood and blood-forming organs and certain disorders involving the immune mechanism; G00-G99: Diseases of the nervous system; ICU: Intensive care units. OI: overall incidence. NICU: neonatal intensive care units. PICU: pediatric intensive care units. WHO: World Health Organization; P00–P96: Certain conditions originating in the perinatal period. *the overall incidence was calculated by the author of this paper, from the sample size and patient with ADR number presented in the study. †the patient with ADR number was calculated by the author of this paper, from the overall incidence and sample size presented in the study. ‡Multivariate analysis. (PDF) [file pone.0182327.s006.pdf]

S6 Table. Comparison between the characteristics of the present cohort study and the characteristics of prospective cohort studies on risk factors for adverse drug reactions in pediatric inpatient.

**Author**	**Andrade et al, 2016**	**Rashed et al, 2014**	**Thiesen et al, 2013**	**Silva et al, 2013**	**Rashed, Neubert, et al, 2012**	**Rashed, Wong, et al, 2012**	**dos Santos and Coelho, 2006**	**Turner et al, 1999**
**Country**	Brazil	China	United Kingdom	Brazil	United Kingdom and Saudi Arabia	Australia, Germany, China, Malaysia and United Kingdom	Brazil	United Kingdom
**Duration of follow-up by research centers**	10 months	4 months	12 months	6 months	3 months	3 months	5 months	3 months
**Total research centers**	1 center	7 centers in the same country	1 center	1 center	2 centers in different countries	5 centers in different countries	1 center	1 center
**Clinical setting**	1 pediatric ward with different specialties	Medical wards, PICU and NICU	17 wards, including oncology wards and the high dependency unit	PICU	Medical wards, PICU and NICU	Paediatric general medical wards	1 pediatric ward with different specialties	5 wards, including cardiac intensive care and ICU
**Method for assessing causality**	Naranjo algorithm	Naranjo algorithm	Naranjo algorithm	Naranjo algorithm	Naranjo algorithm	Naranjo algorithm	WHO definition	Unofficial classification by definition
**ADR included based on assessing causality**	Defined and probable	Defined, probable and possible	Defined and probable	Defined, probable and possible	Defined, probable and possible	Defined, probable and possible	Defined, probable and possible	All the ADR suspected

S7 Table. Continued.

Author	Andrade et al, 2016	Rashed et al, 2014	Thiesen et al, 2013	Silva et al, 2013	Rashed, Neubert, et al, 2012	Rashed, Wong, et al, 2012	dos Santos and Coelho, 2006	Turner et al, 1999
**Data collection**								
Patient record	Yes	Yes	Yes	Yes, to search “triggers”	Yes	Yes	Yes	Yes, only of patient with case notified
Prescription	Yes	Yes	Yes	Yes, to search “triggers”	Yes	Yes	Yes	Yes
Laboratory data	Yes	Yes	Yes, and imaging reports	Yes, to search “triggers”	Yes	Yes	Yes	Yes, only of patient with case notified
Observation of apparent clinical events on the bed	Yes	No	Yes	No	No	No	No	No
Structured interview childrens’ mothers or relatives	Yes	No	No	No	No	No	Yes	No
Discussion with the health care team	Yes	No	No	No	No	No	No	No
“Triggers”	No	No	No	Yes	No	No	No	No
Spontaneous reporting	Yes	No	No	No	No	No	No	Yes
**Risk factor analysis**	Cox Regression	Logistic regression	Cox Regression	Logistic regression	Logistic regression	Logistic regression	Logistic regression	Logistic regression

S7 Table. Continued.

Author	Andrade et al, 2016	Rashed et al, 2014	Thiesen et al, 2013	Silva et al, 2013	Rashed, Neubert, et al, 2012	Rashed, Wong, et al, 2012	dos Santos and Coelho, 2006	Turner et al, 1999
**Sample size**	173	329	5.118	239	737	1115	265	936
**Patients with ADR (OI%)**	38 (21.9%)	9 (2.7%)*	906† (17.7%)	84 (35.1%)	63 (8.5%)	186 (16.7%)	33 (12.5%)	116 (12.4%)*
**Risk factors‡**								
Increase in the number of prescription drugs	Yes (for somnolence)	Yes (≥ 5 drugs)	Yes	No	Yes (≥ 5 drugs)	Yes (1-4 low-risk drugs and 2-3 high-risk drugs)	Yes (6-10 drugs and ≥ 11 drugs)	Yes
Age	No	No	No	Yes (< 48 months)	Yes (6-12 years)	Yes (11-18 years)	No	No
Gender	No	No	No	No	No	No	Yes (male)	No
General anesthetic administration	Yes (for vomiting, nausea, and skin and appendages disorders)	-	Yes	-	-	-	-	-
Oncological treatment	-	-	Yes	-	-	-	-	-
Scheduled admission	-	-	-	-	Yes	-	-	-
Transferred admission	-	-	-	-	Yes	-	-	-
D50-D89	-	-	-	-	-	Yes	-	-
G00-G99	-	-	-	-	-	Yes	-	-
P00–P96	-	-	-	-	-	Yes	-	-

S7 Table. Continued.

Author	Andrade et al, 2016	Rashed et al, 2014	Thiesen et al, 2013	Silva et al, 2013	Rashed, Neubert, et al, 2012	Rashed, Wong, et al, 2012	dos Santos and Coelho, 2006	Turner et al, 1999
Prior history of ADR of the patient	Yes	-	-	-	-	-	-	-
Meglumine antimonate	Yes	-	-	-	-	-	-	-
Antibacterial for systemic use	Yes	-	-	-	-	-	-	-
Antiepileptic drug	Yes	-	-	-	-	-	-	-
Antihistamine drug	Yes (for somnolence)	-	-	-	-	-	-	-
Number of new drugs administered after admission	Yes (for somnolence)	-	-	-	-	-	-	-
Number of intravenous drugs administered	Yes (for somnolence)	-	-	-	-	-	-	-

D50-D89: Diseases of the blood and blood-forming organs and certain disorders involving the immune mechanism; G00-G99: Diseases of the nervous system; ICU: Intensive care units. OI: overall incidence. NICU: neonatal intensive care units. PICU: pediatric intensive care units. WHO: World Health Organization; P00–P96: Certain conditions originating in the perinatal period.

*the overall incidence was calculated by the author of this paper, from the sample size and patient with ADR number presented in the study.

†the patient with ADR number was calculated by the author of this paper, from the overall incidence and sample size presented in the study.

‡Multivariate analysis.
